# Supplementary material for: Efficacy of the Chinese version interpretation bias modification training in an unselected sample: A randomized trial
Source: PLoS One. 2021 Jul 28;16(7):e0255224. doi: 10.1371/journal.pone.0255224 (PMC8318276; doi:10.1371/journal.pone.0255224)
Supplement: S2 File — (DOCX) [file pone.0255224.s003.docx]

**课题研究计划书**

**项目名称：解释偏向与社交焦虑关系及矫正效果研究**

**项目负责人：邓光辉 ，张 帆**

**项目执行人：张 帆**

目 录

[方案摘要 3](#_Toc77233436)

[研究背景 4](#_Toc77233437)

[1. 解释偏向的定义 4](#_Toc77233438)

[2. 解释偏向的研究范式 4](#_Toc77233439)

[3. 解释偏向矫正的研究范式 5](#_Toc77233440)

[4. 解释偏向矫正训练对社交焦虑情绪的改善效果 5](#_Toc77233441)

[5. 不同认知偏向成分的关系 6](#_Toc77233442)

[研究设计 9](#_Toc77233443)

[1. 研究一：军人解释偏向的问卷的编制 9](#_Toc77233444)

[2. 研究二：解释偏向与注意偏向的关系 10](#_Toc77233445)

[3. 研究三：军人消极解释偏向的矫正 12](#_Toc77233446)

[关于手稿和研究方案关系的说明 13](#_Toc77233447)

# 方案摘要

社交焦虑是常见的心理问题之一。通过改善负性解释偏向从而缓解焦虑状态的研究是当前认知偏向研究的热点，但目前研究者对解释偏向产生的机制、解释偏向矫正的焦虑改善作用和作用机制还存在争议，需要进一步研究。在实践需求和研究进展的共同推动下，本课题设计了三个研究，首先开发适用于中国军人的解释偏向问卷，在此基础上探究认知偏向的共同作用理论。最后，借助解释偏向矫正程序探讨改善军人焦虑状态的有效途径，为提高军人和大众心理健康提供新视角和新方法。

# 研究背景

认知偏向（cognitive bias）指个体对某种信息的加工偏好，常见的认知偏向包括注意偏向（attentional bias, AB）、解释偏向（interpretational bias, IB）、记忆偏向（memory bias）及归因偏向（attribution bias）等。其中解释偏向是近年来的研究热点，研究者已开发了多种范式，形成了较为成熟的研究体系。为了对认知偏向进行临床干预，解释偏向矫正（Cognitive bias modification of Interpretation，CBM-I）在解释偏向范式的基础上演化而来，通过大量重复使被试形成某一种方向（积极或者消极）的解释偏向，是目前较为前沿的认知干预方法。

1. 解释偏向的定义

解释是信息加工过程的重要一环，外界信息经过感觉、知觉加工后，我们要为信息赋予意义，这就依靠信息的解释。但是一旦外在信息不明确（如信息模糊或者存在歧义），不同的人可能对其产生完全不同的解释，如对两可图形的解释。对模糊信息的解释还与个体的焦虑水平有关，早在1976年，Beck就提出，焦虑群体对模糊信息存在着系统性的消极解释倾向，到今天，研究者普遍认为这种消极的解释偏向（Interpretational Bias）在焦虑的产生和维持中起到重要作用。解释偏向一般被定义为个体对模糊情景、事件或其他刺激进行负面或威胁解释的倾向^[1]^。

2. 解释偏向的研究范式

解释偏向的研究范式包括以反应时、正确率等指标为因变量的在线测量（on-line measure）和以自我报告结果为因变量的离线测验（off-line measure）。

最为经典的在线测验包括同音异义词范式（Homophone And Homograph Paradigm）、词句联想范式（word sentence association paradigm, WSAP）和以模糊场景为特征的范式。其中，WASP范式首先呈现500ms注视点，之后呈现威胁词或积极词，在词语消失后呈现模糊语句（包括社交情景的和非社交情景的），要求被试判断先前呈现的词语与语句的关系，如果被试认为威胁词与模糊语句相关，则出现了消极的解释偏向。

常用的离线测验主要为模糊故事范式（Ambiguous Vignettes Paradigm）。该范式首先呈现模糊情景，要求被试写下自己的解释或者要求被试从积极、中性及积极解释中做出选择。研究者编制了一些测验进行离线测量，如两可社会情景解释问卷(The Ambiguous Social Situation Interpretation Questionnaire, ASSIQ)，该问卷包括24个项目，其中14个为社交情景，10个为非社交情景，每个情景后有三个选项，分别是对该情景的消极或者中性或者积极解释，要求被试想象当他处在故事中的情景时，对三种解释的可能性进行排序，并对消极解释的出现顺序进行计分，如消极解释最可能出现，则计3分，其次计2分，最次计1分，因此被试得分越高说明其消极解释偏向越明显^[2]^。除了模糊故事范式，为了提升生态效度，还有的研究构建了社交场景，如要求社交焦虑者和非社交焦虑者在一些听众面前进行演讲，之后评价自己的表现，通过比较二者对自身的评价可以发现焦虑者解释偏向的特征。此外，相似性评估任务（Similar Rating Task, SRT）的应用也较为普遍^[3]^。该任务首先呈现模糊情景，阅读后要求被试回答控制问题。之后呈现四个含义明确的句子，其中两个是与情景相关的积极解释和消极解释，称为目标句，另外两个为与情景无关但效价明确的积极和消极句。要求被试判断每个句子与情景的相似性，1分代表非常不同，4分代表非常相似。消极和积极目标句得分的差异作为问卷分数。

3. 解释偏向矫正的研究范式

常见的解释偏向矫正训练又包括Mathews和Mackintosh设计的程序Cognitive Bias Modification of Interpretation（CBM-I）^[4]^和Beard和Amir设计的程序Interpretation Modification Progrem （IMP）^[5]^。CBM-I程序通过引导被试对模糊情景做出积极解释矫正被试的消极解释偏向。该程序一般包括多个模糊情景，每个情景由三个完整句子和一个不完整句子构成，最后的不完整句子缺少某个单词。实验中，程序引导被试为最后一句补充一个积极词，形成积极意义，并借此诱导被试形成积极解释偏向。IMP程序首先向被试呈现威胁词或积极词，然后出现一个模糊语句，要求被试判断词语与语句的关系，当被试赞同积极词或拒绝威胁词时，得到肯定反馈，反之则得到否定反馈。CBM-I和IMP都是依靠反复训练和大量重复使被试的消极认知偏向得到抑制。

4. 解释偏向矫正训练对社交焦虑情绪的改善效果

目前大多数研究支持CBM-I能够改善解释偏向，但对其是否能够改善焦虑症状存在争议。如Nowakowski等人^[6]^对高社交焦虑被试的解释偏向进行了单次CBM-I干预后发现，CBM-I训练能使患者对模糊情景的解释更为积极，但并不能减缓被试在演讲任务中感受到的焦虑。Salemink等人^[7]^对临床样本的研究也发现CBM-I不能改善焦虑症状。相比于CBM-I，使用IMP干预程序进行的研究似乎得到了更好的结果，如Beard和Amir^[8]^发现，在进行了8次的IMP训练后，高社交焦虑大学生对模糊情景的积极解释增加、消极解释减少，自我报告的焦虑水平显著下降。该课题组在社交焦虑临床样本中重复了这一研究结果^[9]^。相比于CBM-I，IMP的训练时间更长、次数更多。这可能是IMP训练更为有效的原因，但目前使用IMP的研究相对较少，该程序的有效性还需要更多研究来证实。

5. 不同认知偏向成分的关系

有研究者提出，不同认知偏向之间应该是相互联系的，这种相互联系使他们共同引起了适应不良反应^[10]^。注意偏向与解释偏向作为焦虑障碍的重要特征，二者的关系如何？当前研究者试图通过认知偏向矫正程序改变注意或解释偏向其中之一，从而判断一种认知偏向的改变对另一种的影响。从信息加工过程的角度来看，注意偏向反映的是对威胁的早期加工，解释偏向反映的则是后期加工过程，因此注意偏向会对解释偏向产生影响^[11]^。遵循这一思路，一些研究者对焦虑人群的注意偏向进行了干预并观察其解释偏向的变化。Bowler等人^[12]^对自我报告有焦虑情绪的大学生分别进行了为期8周的ABM和CBM-I训练，结果发现，接受ABM训练的大学生，其消极注意和解释偏向均有所改善；但接受CBM-I的训练组，只有解释偏向的改善，却仍保持着消极的注意偏向，说明注意偏向影响解释偏向，但并没有相反的影响。White等人^[13]^训练被试关注消极的注意目标，发现相比于对照组，干预组对模糊情景的解释也更为消极，同样说明注意偏向影响解释偏向。但也有研究发现影响的方向是相反的，即干预解释偏向能对注意偏向产生影响。Amir等人^[14]^对有社交焦虑的正常人进行了单次CBM-I训练，结果发现相比于对照组，干预组的解释偏向和注意偏向均有显著下降，说明解释偏向影响注意偏向。总之，目前的研究确实发现注意和解释偏向存在一定的联系，但对它们相互作用的方向还不明确。

**参考文献**

[1] Lee J S, Mathews A, Shergill S, Yiend J. Magnitude of negative interpretation bias depends on severity of depression[J]. Behaviour Research & Therapy, 2016, 83: 26-34.

[2] Stopa L, Clark D M. Social phobia and interpretation of social events[J]. Behaviour Research & Therapy, 2000, 38(3): 273-283.

[3] Yiend J, Lee J S, Tekes S, Atkins L, Mathews A, Vrinten M, et al. Modifying Interpretation in a Clinically Depressed Sample Using ‘Cognitive Bias Modification-Errors’: A Double Blind Randomised Controlled Trial[J]. Cognitive Therapy & Research, 2014, 38(2): 146-159.

[4] Mathews A, Mackintosh B. Induced emotional interpretation bias and anxiety.[J]. J Abnormal Psychol, 2000,109(4):602-615.

[5] Beard C, Amir N. A multi-session interpretation modification program: Changes in interpretation and social anxiety symptoms[J]. Behav Res Ther, 2008,46(10):1135-1141.

[6] Nowakowski M E, Antony M M, Koerner N. Modifying interpretation biases: Effects on symptomatology, behavior, and physiological reactivity in social anxiety[J]. J Behav Ther Exp Psychiatry, 2015,49(Pt A):44-52.

[7] Salemink E, Kindt M, Rienties H, et al. Internet-based cognitive bias modification of interpretations in patients with anxiety disorders: a randomised controlled trial.[J]. J Behav Ther Exp Psychiatry, 2014,45(1):186-195.

[8] Amir N, Taylor C. Interpretation Training in Individuals With Generalized Social Anxiety Disorder: A Randomized Controlled Trial[J]. J consult clin psychol, 2012,80:497-511.

[9] Beard C, Amir N. A multi-session interpretation modification program: Changes in interpretation and social anxiety symptoms[J]. Behav Res Ther, 2008, 46(10): 1135-1141.

[10] Everaert J, Duyck W, Koster E H W. Attention, interpretation, and memory biases in subclinical depression: A proof-of-principle test of the combined cognitive biases hypothesis.[J]. Emotion, 2014, 14(2): 331-340.

[11] Muris P, Field A P. Distorted cognition and pathological anxiety in children and adolescents.[J]. Cognition Emotion, 2008, 22(3): 395-421.

[12] Bowler J O, Hoppitt L, Illingworth J, Dalgleish T, Ononaiye M, Perezolivas G, et al. Asymmetrical transfer effects of cognitive bias modification: Modifying attention to threat influences interpretation of emotional ambiguity, but not vice versa[J]. J Behav Ther Exp Psychiatry, 2016, 54: 239.

[13] White L K, Suway J G, Pine D S, Bar-Haim Y, Fox N A. Cascading effects: The influence of attention bias to threat on the interpretation of ambiguous information[J]. Behav Res Ther, 2011, 49(4): 244.

[14] Amir N, Bomyea J, Beard C. The effect of single-session interpretation modification on attention bias in socially anxious individuals.[J]. J Anxiety Disorders, 2010, 24(2): 178-182.

# 研究设计

1. 研究一：军人解释偏向的问卷的编制

**被试：**军人，N≈1000。

**工具：**1、自编军人解释偏向问卷（改编自两可社会情景解释问卷，根据军人模糊情景库重新设计模糊情景；参考：Zhang F, Zhang X, Mao X, Chen A, Yin Q, Deng G. Interpretation bias of high trait anxiety Chinese military servicemen in ambiguous military scenarios. Medicine[J]. 2020,99(3):e18746.）；2、校标工具：a：负性认知加工偏向问卷（来源：赖薇,郑飞宇,谢守蓉,李晋,李丽,王立菲,赵梦雪,刘云波,蒋娟,王佳,徐媛媛,徐文佳,冯正直.不同海拔高度军人焦虑特点与其负性认知偏向的关系[J].第三军医大学学报,2017,39(15):1525-1531.）；b：特质焦虑量表（STAI-T）；C：交往焦虑量表-IAS

**数据收集：**采用随机抽样法，在基层部队发放问卷和回收自编问卷和校标问卷

**统计方法：**内部一致性信度、差异检验（区分效度）、相关分析（校标关联效度）

**预期结果：**高低焦虑军人解释偏向得分差异显著；负性认知总分与解释偏向相关

2. 研究二：解释偏向与注意偏向的关系

**被试：**军人，N≈60。

**工具：**1、注意偏向AB的测量：注意偏向的测量采用点探测任务（dot probe paradigm）。实验程序来自TAU-NIMH ABMT 程序包(<http://people.socsci.tau.ac.il/mu/anxietytrauma/research/>)，改编成中文材料。实验过程通过E-prime程序执行，见图1。AB=刺激出现在中性词后的平均反应时（威胁-不一致反应时）-刺激出现在消极词后的平均反应时（威胁-一致反应时）。2、解释偏向IB的测量：研究一自编问卷。3、情绪的测量：抑郁-焦虑-压力量表简体版-DASS-21、IAS。


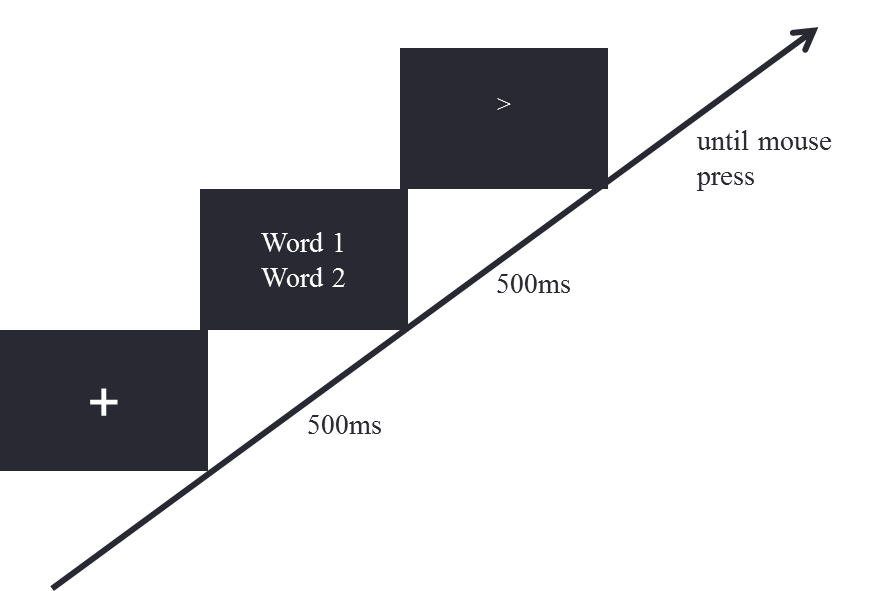


图1 注意偏向测量程序

**眼动记录方法：**使用 Tobii-TX300红外眼动仪采集被试的眼动数据，设备采样率为300Hz。精度标准0.14度，最大扫视角度35度，实验过程中要求被试尽量保持头部竖直，眼睛与眼动仪的距离保持在60cm。实验的每个试次单独呈现在一屏上，电脑液晶屏幕为17英寸，屏幕分辨率1280×1024像素。参考前人的处理方式，采用50ms及以上的停留作为一次注视，使用双眼平均后的眼动数据。主要指标为被试在点探测任务中观察中性词和威胁词的凝视时间、总注视时间和注视次数。

**过程：**根据研究一的结果，选取社交焦虑在前30%和后30%的两组被试各30人。测量高、低社交焦虑组被试的注意和解释偏向，其中，在注意偏向的测量中，收集眼动数据。

**数据分析：**参考以往研究，将眼动数据划分为200ms前的眼动和200-500ms之间的眼动，作为因变量AB_200_和AB_200-500_，另一个因变量为解释偏向问卷分数IB。以AB_200_和AB_200-500_为因变量，采用2（高社交焦虑/低社交焦虑）×2（中性词/威胁词）对因变量进行混合因素方差分析。以IB为自变量，分析高/低社交焦虑被试的解释偏向差异。最后通过Pearson积差相关分析三个因变量之间的相关。

**预期结果：**对200ms内的眼动指标，组别×词性的交互作用显著，高社交焦虑被试对威胁刺激的注意次数、注意时间和凝视时间均高于低人际焦虑被试，表现出早期注意警觉；对200ms-500ms内的眼动指标，组别×词性的交互作用显著，高社交焦虑被试对威胁刺激的注意次数、注意时间和凝视时间均低于低人际焦虑被试，表现出注意回避。AB_200_，AB_200-500_和IB之间均存在显著相关，支持认知偏向联合作用假说。

3. 研究三：军人消极解释偏向的矫正

**被试：**军人，N=60

**工具：**对注意偏向和解释偏向的测验同前。

**训练过程：**采用解释偏向矫正程序（Interpretation modification program，IMP）。相同的IMP训练连续执行五天，在训练第一天和最后一天收集注意偏向、解释偏向和社交焦虑数据。采用2（测验时间：前/后）×2（组别：对照组/训练组）混合实验设计，具体过程见图2。


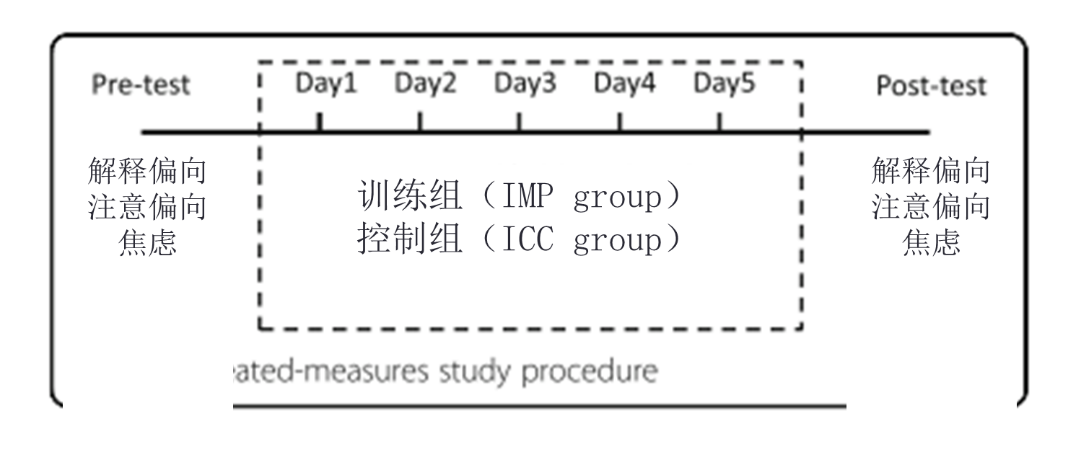


**图2 随机对照实验设计图**

**数据分析：**参照以往研究，通过点探测任务获得前/后测注意偏向指标AB1和AB2，以前后测解释偏向问卷分数和社交焦虑问卷分数为IB1、IB2及IAS1、IAS2。分别以这些指标为因变量进行2×2方差分析。同时通过独立样本t检验比较前后测中，对照组和实验组分数的差异。

**预期结果：**训练×时间交互作用显著，训练组在训练后焦虑水平明显下降，控制组没有改变；两组前测的因变量无显著差异，在后测中，训练组因变量指标显著下降。说明IMP训练有效。

# 关于手稿和研究方案关系的说明

1、这个实验设计是研究执行人博士论文的实验设计。

2、手稿中呈现的实验结果，是本研究设计中实验三的结果。研究执行过程基本上执行了研究设计，但限于部队管理要求和新冠肺炎疫情影响，我们没有到部队采样，而是使用了学生样本。

3、本研究设计没有在任何地方公开发表，使用请标明出处。
