# Supplementary material for: Efficacy of the Chinese version interpretation bias modification training in an unselected sample: A randomized trial
Source: PLoS One. 2021 Jul 28;16(7):e0255224. doi: 10.1371/journal.pone.0255224 (PMC8318276; doi:10.1371/journal.pone.0255224)
Supplement: S3 File — (DOCX) [file pone.0255224.s004.docx]

**Project Protocol**

**Project Name: Study on the relationship between interpretation bias and social anxiety and its correction effect**

**Project leader: Guanghui Deng, Fan Zhang**

**Project executive: Fan Zhang**

contents

[Abstract 3](#_Toc77233088)

[Background 4](#_Toc77233089)

[1. Definition of interpretation bias 4](#_Toc77233090)

[2. Paradigms of interpretation bias 4](#_Toc77233091)

[3. Paradigms of interpretation bias modification 5](#_Toc77233092)

[4. The improvement effect of bias correction training on social anxiety 6](#_Toc77233093)

[5. The combined cognitive bias hypothesis 6](#_Toc77233094)

[Methods 10](#_Toc77233095)

[Study 1：Development of interpretation bias for military personnel 10](#_Toc77233096)

[Study 2: A test of the combined cognitive biases hypothesis 11](#_Toc77233097)

[Study 3: Modification of negative interpretation bias in military personnel 13](#_Toc77233098)

[A note on the relationship between the manuscript and the study protocol 14](#_Toc77233099)

# Abstract

Social anxiety is one of the most common psychological problems. Alleviating social anxiety by improving negative interpretation bias is a hot spot in current cognitive bias research. However, there are still controversies on the mechanism of interpretation bias, the improvement effect and mechanism of interpretation bias correction and further researches are needed. With the needs of theory and practice, the present research protocol designed three studies. Firstly, the study would develop an interpretation bias questionnaire applicable to Chinese soldiers, and then the combined cognitive bias hypothesis would be explored. Finally, the efficiency of improving social anxiety by interpretive bias correction procedure would be tested. The present study would provide a new perspective and method to improve the mental health in general public.

# Background

Cognitive bias refers to the biases in information processing, common cognitive biases include attentional bias (AB), interpretation bias (IB), memory bias and attribution bias etc. Among them, interpretation bias has become a research hotspot in recent years. Researchers have developed a variety of paradigms and formed a relatively mature research system. In order to perform clinical intervention with Cognitive bias, Cognitive bias Modification of Interpretation (CBM-I) has been adapted from Interpretation bias paradigm. CBM-I corrects subjects’ interpretation bias by a lot of repetition of interpretation (positive or negative) in a certain direction, which is one of the most advanced cognitive intervention methods.

1. Definition of interpretation bias

Interpretation is an important part of information processing. After the external information is processed by perception and perception, we should give meaning to the information, which depends on the interpretation of information But once the external information is not clear (such as ambiguous information), different people may interpret it in completely different ways. Interpretation of ambiguous information is associated with the anxiety level of the individual. As early as 1976, A.T. Beck put forward that anxiety group had systemic negative interpretation of ambiguous information. So far, researchers is generally believed that the negative Bias (Interpretational Bias, IB) play an important role in development and maintenance of anxiety. IB is generally defined as an individual's tendency to interpret ambiguous situations, events, or other stimuli negatively or threateningly[1].

2. Paradigms of interpretation bias

Paradigms of IB include on-line measures based on reaction time and correction rates and off-line measures based on self-report results.

The most classic on-line measures includes Homophone And Homograph Paradigm, word sentence association paradigm (WSAP) and paradigm based on ambiguous scenarios. In WASP, a fixation is presented for 500ms, and then a threatening/non-threatening word is presented which is followed by an ambiguous sentence. The participants would be asked to judge linkage between the sentence and the word, and if they think the threatening word is associated with the sentence, they show negative interpretation bias; or, they do not have interpretation bias.

The most common off-line measure is the Ambiguous Vignettes Paradigm. In the paradigm, a vague situation in which participants are asked to write down their own explanations or to choose between positive, neutral or positive explanations. Researchers has created an Ambiguous Social Situation Interpretation Questionnaire (ASSIQ), containing 24 items (14 of which are social situations, 10 of which are non-social situations), and each item is followed by three alternations: one positive interpretation, one negative interpretation and one neutral interpretation. The participants are asked to rank the likelihood of the three explanations, and score the order in which the negative explanation appears[2]. In addition to the Ambiguous Vignettes Paradigm, researchers also construct social scenes in order to improve the ecological validity. For example, social anxiety participants and non-social anxiety participants are asked to speech in front of some of the audience and self-evaluate their own performance. Bu comparing the two evaluation between social and non-social anxiety groups, negative interpretations are very clear. In addition, the similarity assessment tasks (Similar Rating Task, SRT) applications are also commonly used[3]. The task first presents an ambiguous scene and then asks the subjects to answer control questions after reading. Then four sentences with clear meaning are presented, two of which are positive and negative explanations related to the situation, called the target sentences, and the other two are positive and negative sentences unrelated to the situation but with clear valences. Participants are asked to judge how similar each sentence is to the situation.

3. Paradigms of interpretation bias modification

Common paradigms of interpretation bias modification include Cognitive Bias Modification of Interpretation (CBM-I) designed by Mathews and Mackintosh [4] and Interpretation Modification Progrem (IMP) designed by Beard and Amir[5]. CBM-I program corrects the negative interpretation bias of the subjects by guiding them to make positive interpretations of the ambiguous situations. The program typically consists of several ambiguous scenarios, each consisting of three complete sentences and one incomplete sentence, with the final incomplete sentence missing a word. During correction, the program guided the subjects to add a positive word to the last sentence to form a positive meaning, and thereby guiding the subjects to form a positive interpretation bias. The IMP program first presents a threat word or a positive word to the subjects, and then presents an ambiguous statement, asking the subjects to judge the relationship between the words and the statement. Once the subjects agree with the positive word or reject the threat word, they will get positive feedback, and otherwise they will get negative feedback. The common feature of CBM-I and IMP is that they both correction negative interpretation bias by a lot of repetition.

4. The improvement effect of bias correction training on social anxiety

At present, most studies support that CBM-I could improve interpretation bias, but whether it can improve anxiety symptoms is controversial. For example, Nowakowski et al. [6] conducted a single CBM-I intervention in subjects with high social anxiety and found that CBM-I training could make patients interpret ambiguous situations more positively, but could not alleviate the anxiety that subjects felt during the following speech task. Salemink et al. [7] also found that CBM-I could not improve anxiety symptoms in clinical samples. Compared with CBM-I, studies using IMP intervention procedures seem to have better results. For example, Beard and Amir[8] found that after 8 IMP training sessions, the positive interpretation of ambiguous situations were increased and the negative interpretation of ambiguous situations were decreased, and the self-reported anxiety level was significantly reduced among students with high social anxiety. The same research group repeated this result in the clinical sample of social anxiety [9].Compared to CBM-I, IMP training is longer and this may be the reason why IMP training is more effective, but there are relatively few studies using IMP and more studies are needed to confirm the effectiveness of the program.

5. The combined cognitive bias hypothesis

Some researchers have proposed that different cognitive biases should be related to each other and jointly cause adverse adaptive reactions [10]. Attentional bias and explanation bias both are important characteristics of anxiety disorder. From the perspective of information processing, attention bias reflects the early processing of threats, while interpretation bias reflects the late processing, so attention bias might have an impact on interpretation bias [11]. Following this line of thinking, some researchers have intervened attentional bias of anxious people and observed changes in its interpretive bias. Bowler et al. [12] conducted ABM and CBM-I training for 8 weeks on college students with self-reported anxiety, and found that the negative attention and interpretation bias of college students receiving ABM training were improved. However, in the training group receiving CBM-I, only interpretation bias was improved, negative attention bias remained, indicating that attention bias affected the interpretation bias, but did not vice versa. White et al. [13] trained subjects to pay attention to negative attention targets and found that compared with the control group, the interpretation of ambiguous scenes in the intervention group was also more negative, which also indicated that attention bias influenced interpretation bias. However, some studies have found that the influence direction is opposite, that is, intervention interpretation bias could have an impact on the attention bias. Amir et al. [14] conducted a single CBM-I training for normal people with social anxiety, and found that the interpretation and attention bias in the intervention group were significantly decreased compared with the control group, indicating that change in interpretation bias affects attention bias. In summary, current researches indeed find a connection between attention and explanatory bias, but the direction of their interaction is unclear.

**References**

[1] Lee J S, Mathews A, Shergill S, Yiend J. Magnitude of negative interpretation bias depends on severity of depression[J]. Behaviour Research & Therapy, 2016, 83: 26-34.

[2] Stopa L, Clark D M. Social phobia and interpretation of social events[J]. Behaviour Research & Therapy, 2000, 38(3): 273-283.

[3] Yiend J, Lee J S, Tekes S, Atkins L, Mathews A, Vrinten M, et al. Modifying Interpretation in a Clinically Depressed Sample Using ‘Cognitive Bias Modification-Errors’: A Double Blind Randomised Controlled Trial[J]. Cognitive Therapy & Research, 2014, 38(2): 146-159.

[4] Mathews A, Mackintosh B. Induced emotional interpretation bias and anxiety.[J]. J Abnormal Psychol, 2000,109(4):602-615.

[5] Beard C, Amir N. A multi-session interpretation modification program: Changes in interpretation and social anxiety symptoms[J]. Behav Res Ther, 2008,46(10):1135-1141.

[6] Nowakowski M E, Antony M M, Koerner N. Modifying interpretation biases: Effects on symptomatology, behavior, and physiological reactivity in social anxiety[J]. J Behav Ther Exp Psychiatry, 2015,49(Pt A):44-52.

[7] Salemink E, Kindt M, Rienties H, et al. Internet-based cognitive bias modification of interpretations in patients with anxiety disorders: a randomised controlled trial.[J]. J Behav Ther Exp Psychiatry, 2014,45(1):186-195.

[8] Amir N, Taylor C. Interpretation Training in Individuals With Generalized Social Anxiety Disorder: A Randomized Controlled Trial[J]. J consult clin psychol, 2012,80:497-511.

[9] Beard C, Amir N. A multi-session interpretation modification program: Changes in interpretation and social anxiety symptoms[J]. Behav Res Ther, 2008, 46(10): 1135-1141.

[10] Everaert J, Duyck W, Koster E H W. Attention, interpretation, and memory biases in subclinical depression: A proof-of-principle test of the combined cognitive biases hypothesis.[J]. Emotion, 2014, 14(2): 331-340.

[11] Muris P, Field A P. Distorted cognition and pathological anxiety in children and adolescents.[J]. Cognition Emotion, 2008, 22(3): 395-421.

[12] Bowler J O, Hoppitt L, Illingworth J, Dalgleish T, Ononaiye M, Perezolivas G, et al. Asymmetrical transfer effects of cognitive bias modification: Modifying attention to threat influences interpretation of emotional ambiguity, but not vice versa[J]. J Behav Ther Exp Psychiatry, 2016, 54: 239.

[13] White L K, Suway J G, Pine D S, Bar-Haim Y, Fox N A. Cascading effects: The influence of attention bias to threat on the interpretation of ambiguous information[J]. Behav Res Ther, 2011, 49(4): 244.

[14] Amir N, Bomyea J, Beard C. The effect of single-session interpretation modification on attention bias in socially anxious individuals.[J]. J Anxiety Disorders, 2010, 24(2): 178-182.

# Methods

Study 1：Development of interpretation bias for military personnel

**subjects：**military personnel, N≈1000

**Measurements:** 1. Interpretation bias for military personnel (adapted from The Ambiguous Social Situation Interpretation Questionnaire, ASSIQ, ambiguous situations would be replaced by ambiguous military scenarios (reference: Zhang F, Zhang X, Mao X, Chen A, Yin Q, Deng G. Interpretation bias of high trait anxiety Chinese military servicemen in ambiguous military scenarios. Medicine[J]. 2020,99(3):e18746.) ; 2. Criteria validity: a. Negative cognitive processing bias questionnaire (see Lai W et al. Relationship between anxiety characteristics of soldiers at different altitudes and their negative cognitive bias [J]. Journal of the Third Military Medical University, 2017,39(15):1525-1531[in Chinese].); b: Trait Anxiety Scale (STAI-T); c: Social Anxiety Scale -IAS

**Data collection:** Random sampling method was adopted to distribute questionnaires in the grass-roots units

**Data analyses:** reliability-Cronbach’s ａ; Validity- discrimination validity (independent-t test between high and low social anxiety group) and criterion-related validity (Pearson’s correlation between the interpretation bias for military personnel questionnaire and negative cognitive processing bias questionnaire, STAI-T and IAS)

**Expected results:** Cronbach’s ａof the interpretation bias for military personnel questionnaire is high (>0.7); and significant differences in scores of the interpretation bias for military personnel questionnaire between high and low social anxiety personnel would be found; significant correlations between self-developed scale and the referenced scales would be found.

Study 2: A test of the combined cognitive biases hypothesis

**subjects：**military personnel, N≈60

**Measurements:** 1. Measurement of attentional bias: using dot-probe paradigm to measure attentional bias. The experimental program would be adapted from the Tau-Nimh ABMT package. Stimulations in the program would be translated into Chinese. The experimental program would be implemented through E-Prime. See Figure 1 for a trial in dot-probe. 2. Measurement of interpretation bias: the interpretation bias for military personnel questionnaire in study 1. 3. Measurement of (social) anxiety: IAS and Depression-Anxiety-Stress scale, DASS-21.


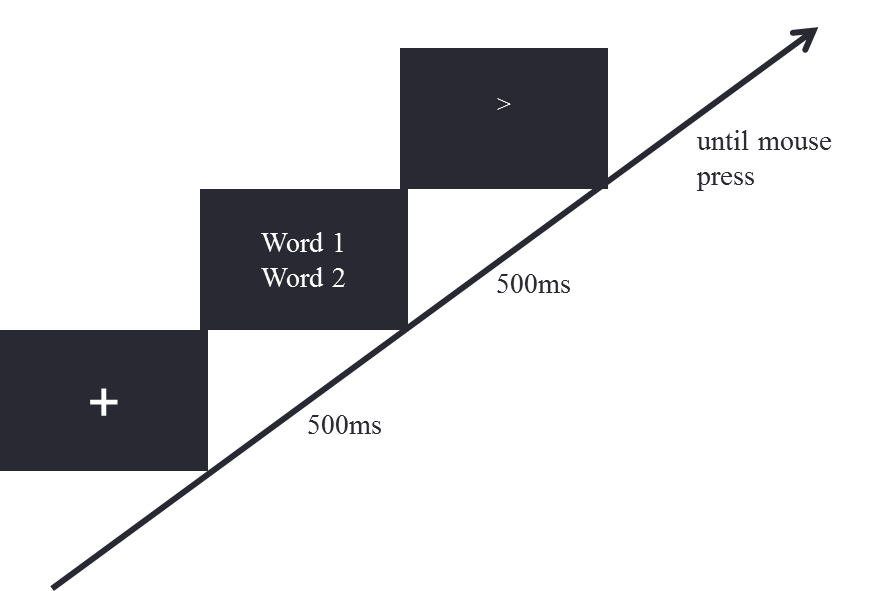


Figure 1 Sequence of events in the dot-probe task

**Eye-movement:** Tobii-tx300 infrared eye tracker would be used to collect the eye movement data of the subjects, and the sampling rate of the device was 300Hz.The accuracy standard was 0.14 °, and the maximum sash Angle was 35 °. During the experiment, subjects were required to keep their heads upright, and keep the distance between their eyes and the eye tracker at 60cm. Each trial of the experiment was presented on a 17-inch LCD screen with a resolution of 1280×1024 pixels. Referring to the previous studies, a stay of 50ms or more was used as a fixation, and the eye movement data after the average binocular level was used. The main outcomes would be the gaze time, total fixation and total fixation counts in the two area of interesting (neutral words verse threat words) in the dot-probe task.

**Procedure:**According to the results of study 1, 30 subjects in the first and 30 in the last 30% of social anxiety in participants in study 1were selected. The attention and interpretation bias of subjects in the high and low social anxiety groups were measured, and eye movement data were collected during dot-probe task.

**Data analyses:** Referring to previous studies, eye movement data were divided into eye movement before 200ms and eye movement between 200-500ms and they are defined as dependent variables: AB_200_ and AB_200-500_. Another dependent variable is the interpretation bias questionnaire score: IB. With AB_200_ and AB_200-500_ as dependent variables, 2 (group: high social anxiety/low social anxiety) ×2 (word class: neutral word/threat word) mixed factor ANOVA would be conducted. IB was used as independent variable to analyze the differences of interpretation bias in high/low social anxiety subjects. Finally, the correlations between the three dependent variables was analyzed by Pearson’s correlation analyses.

**Expected results:** For eye movement within 200ms, the interaction between groups × word class was significant. Subjects with high social anxiety would show more fixations, fixation duration and gaze duration to threatening stimuli than those with low interpersonal anxiety, showing early attention vigilance. For eye movement within 200ms-500ms, the interaction of group × part of speech would be significant, and the fixations, fixation duration and gaze duration of subjects with high social anxiety would be lower than those with low interpersonal anxiety, showing attention avoidance. There would be significant correlations between AB_200_, AB_200-500_and IB, supporting the hypothesis of combined cognitive bias.

Study 3: Modification of negative interpretation bias in military personnel

**Subjects:** military personnel, N=60

**Measurements:** The tests for attentional bias, interpretive bias and social anxiety are the same as study 2.

**Procedure:** The same IMP training was performed for five consecutive days, and data on attentional bias, interpretive bias, and social anxiety were collected on the first and last day of the training. A mixed experimental design of 2 (test time: before/after) ×2 (group: control group/training group) would be adopted. See Figure 2 for the procedure.


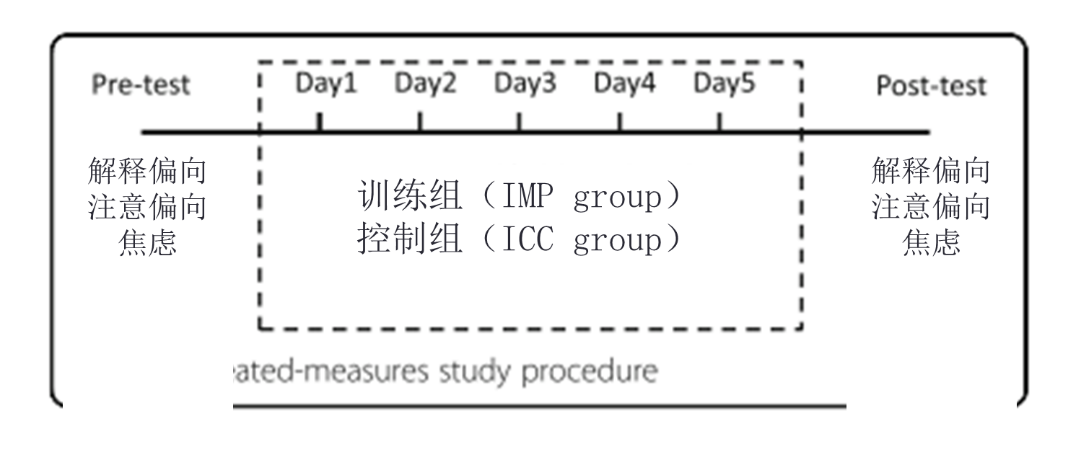


**Figure 2 Procedure in study 3**

**Data analysis:** Referring to previous studies, the pre-training/after-training attention bias indicators AB1 and AB2 would be obtained by dot-probe task. The scores of the pre-training/ after-training interpretation bias questionnaire and the social anxiety questionnaire were IB1, IB2, IAS1 and IAS2. 2×2 analysis of variance would be carried out with these indicators as dependent variables respectively. At the same time, independent sample T test was used to compare the scores of the control group and the experimental group before and after training.

**Expected results:** The training × time interaction would be significant, the anxiety level of the training group decreased significantly after the training, and the control group did not change. There would be no significant difference in the dependent variables between the two groups in the pre-test, while in the post-test, the dependent variables in the training group significantly decreased. These results indicates that the IMP training is an effective way to reduce social anxiety.

# A note on the relationship between the manuscript and the study protocol

1. This project protocol is the study design of the research executor's doctoral thesis.

2. The results presented in the manuscript are the results of study 3 in the project protocol. The study implementation process basically implemented the study design, but limited to the management requirements in military and the impact of the COVID-19 outbreak, we did not go to the troops to collect data, but instead used student samples.

3. The project protocol has not been published anywhere, please indicate the source of use.
