# Supplementary material for: Efficacy of the Chinese version interpretation bias modification training in an unselected sample: A randomized trial
Source: PLoS One. 2021 Jul 28;16(7):e0255224. doi: 10.1371/journal.pone.0255224 (PMC8318276; doi:10.1371/journal.pone.0255224)
Supplement: Attachment — Submitted filename: response to reviewers.docx [file pone.0255224.s005.docx]

**Response to reviewers**

Dear editors and reviewers,

Thank you for your helpful suggestions! After careful study of the comments, we revised the original paper carefully according to the following suggestions. The revised parts were marked in red in the related manuscript. And here are the reviewers’ comments and our reply.

Reviewer #1: The objective of this prospective, randomized controlled trial (RCT) is to assess the effectiveness of the IMP training (compared to the ICC training) on evaluation of interpretation bias, social anxiety, and attentional bias, and subsequently determine possible mediation effects. The study was registered as a RCT within the Chinese Clinical Trial Registry (with a legit ChiCTR number), and was approved by the respective IRB/Ethics Committee. While the study objectives sound interesting, is important, and on target, some shortcomings were observed, in regards to abiding by the CONSORT guidelines for conducting and reporting results of high-quality randomized controlled trials (RCTs). Some other (statistical) comments were also provided.


1. Methods:

Methods reporting appeared very messy. An orderly manner is suggested, following CONSORT guidelines, without repeating information, such as Trial Design, Participant Eligibility Crtieria and settings, Interventions, Outcomes, sample size/power considerations, Interim analysis and stopping rules, Randomization (details on random number generation, allocation concealment, implementation), Blinding issues, etc, should be mentioned. The authors are advised to create separate subsections for each of the possible topics (whichever necessary), and that way produce a very clear writeup. I see the Authors indeed made an attempt; however, they are advised to write it carefully, following nice examples in the manuscript below:
https://www.sciencedirect.com/science/article/pii/S0889540619300010

- Answer:

Thank you for the advice. We had read the recommended paper carefully and reorganized the statements in the methods part. Subtitles were also added. Please check the revised part in the methods.

Specific comments:
(a) For instance, the randomization and allocation concealment should be made very clear (they are NOT the same thing); the trial staff recruiting patients should NOT have the randomization list. Randomization should be prepared by the trial statistician, and he/she would not participate in the recruiting.

- Answer:

Randomization and allocation concealment was revised as following:

*2.7 Randomization and blinding*

*Randomization in 1:1 allocation was determined by the following process: upon arriving at the laboratory, participants were informed of their numbers which were generated automatically by the computer. The participants and the trial staffs (SL and ZY) know the number but they were blind to the allocation. In fact, if the number was an odd number, the participant was assigned to the ICC group and if the number was an even number, the participant was assigned to the IMP group. Allocation concealment was kept until the end of the study.*

(b) The paper states: "....assigned to IMP and ICC groups with 1:1 allocation ratio." (see page 4). Was it a block randomization (BR), given that BR is often recommended in clinical studies to ensure a balance in sample size across groups over time.
https://www.ncbi.nlm.nih.gov/pmc/articles/PMC2267325/
If Yes, then what's the block size?

- Answer: This was not a block randomization; it was just a simple randomization without blocks. I think the original description of randomization was not clear and please see: *2.7 Randomization and blinding* for the revised description.

(c) Sample size/power: The sample size/power computations should be conducted using the primary response; it is not clear what was done. Furthermore, the sample size/power statement should include "the name of the statistical test (one- or two-sided) employed", and also the sample size number thus obtained. Those are missing.

- Answer: we added “2.5 Sample size calculation” to explain the sample size calculation details. Sample size was based on detecting changes in social anxiety levels, with the assumptions of a two-sided 5% significance level. To detect a large interaction effect size (0.4), the total sample size would be 52 to give a power of 80%.

(d) Statistical Analysis: Overall, looks straightforward. Repeated measures ANOVA was used; however, ANOVA methods are based on strict normality of the responses. Was that ascertained in the analysis?

Multiple imputation was conducted, but what method was used? Was it MICE?
https://www.ncbi.nlm.nih.gov/pmc/articles/PMC3074241/

- Answer:

First, the Multivariate imputation by chained equations was used to address missing values. We added the name the of the method in Data analysis.

Secondly, to test the normality of the data, Shapiro-Wilk tests were conducted and the results revealed that all the variables were consist with normal distribution except for DASS_A score. Results showed that Z=0.16, p<0.01 for pre-training DASS_A; Z=0.14, p<0.05 for post-training DASS_A. We tried several methods to normalize the data and found that reciprocal works. Z=0.11, p=0.16 for reciprocal of DASS_A before training, and Z=0.11, p=0.09 for reciprocal of DASS_A after training. Reciprocals of DASS_A were entered into ANOVA and relevant results were revised.

2. Results & Conclusions:

(a) The authors should check that any statement of significance should be followed by a p-value in the entire Results section. The Results section look OK.

- Answer:

The results section was checked.

(b) The current study utilizes 51 patients, almost giving the feeling of a pilot study. The Conclusions section should clearly state that the results/conclusions are "only" from this Chinese population, and allude to future studies with higher sample sizes, and/or combining other populations to determine the differences.

- Answer:

First, the limitation of small sample size was added in the last paragraph of discussion.

Secondly, the conclusion section was revised in consideration of the small sample size.

Reviewer #2: This article reports an RCT of an interpretation training program vs. sham training on social anxiety in an unselected Chinese student sample.

Overall, my evaluation of the manuscript was positive. It represents a first step in testing out this particular interpretation training paradigm for social anxiety in a Chinese sample, as is reported appropriately as such. The reporting standards are generally good, and other strengths include the pre-registration, availability of data and protocol, and use of intention to treat analyses. I have only some relatively minor suggestions to make:

Abstract:

Say 'may be an effective way' instead of 'is an effective way' in the first sentence -efficacy is still an open question
2nd sentence needs reworking or some punctuation added to be easily understandable

- Answer:

Abstract of the manuscript was revised.

Introduction:
The first paragraph needs to be a bit more cautious about the evidence for CBM, as it is a bit mixed.

- Answer:

The original sentence was modified as “Meta analyses of Randomized Controlled Trials (RCT) have provided early evidence for ABM and CBM-I in reducing cognitive biases and social anxiety symptoms, but the effects were moderated by characteristics of the training procedure and study design”.

Methods:
Randomization - I appreciated the detail provided by the authors, but it was still is a bit unclear. Was the allocation actually random (and if so how was it done - e.g. any blocks / stratification etc) or was it simply pseudo-random based on alternating participant numbers?

- Answer:

Following this advice and the suggestion made by reviewer 1, the methods section was reorganized. And a new subtitle “2.7 Randomization and blinding” was added to describe the randomization and concealment. Randomization in 1:1 allocation was determined by the following process: upon arriving at the laboratory, participants were informed of their numbers which were generated automatically by the computer. The participants and the trial staffs (SL and ZY) know the number but they were blind to the allocation. In fact, if the number was an odd number, the participant was assigned to the ICC group and if the number was an even number, the participant was assigned to the IMP group. Allocation concealment was kept until the end of the study.

What were the predictor variables for the multiple imputation, and how many imputed datasets were created?

- Answer:

Replacement values were generated for all dependent variables using multivariate imputation by chained equations based on five replications. See 2.8 data analysis for the revised description.

Results:
When describing the results for the DASS it would be useful to have the statistics and the effect sizes for the non-significant main effect of time and the timex group interaction. Given that the group x time interaction is n.s. it is not really justified to present the two t-tests comparing pre and post-training scores across the groups.

- Answer:

T-tests for n.s. interaction effects were deleted.

Discussion:
There are a couple of recent studies investigating different kinds of CBM for interpretation in Chinese samples, and it would be good to refer to these in the context of the relatively recent testing of CBM-I in China:
https://link.springer.com/article/10.1007/s12144-020-01094-4
<https://link.springer.com/article/10.1007/s12144-020-00867-1>

<https://pubmed.ncbi.nlm.nih.gov/28855880/>

- Answer:

Thank you for recommending the above papers. We had read them carefully and cited them. These works showed the effectiveness of CBM-I in Chinese with depression or social anxiety symptoms. Although Yang et al. (2017) found that only CBM-I group showed less threat interpretation and more benign interpretation than control after training, they did not report change in social anxiety. And the contribution of our work was that our work extended these previous studies by showing that utilizing a Chinese version IMP can modify interpretation bias and social anxiety in healthy adults. See the revised sentences in the first paragraph of discussion section.

Reviewer #3: The authors test the efficacy of a Chinese version of interpretation bias modification training on interpretation bias, attention bias, and self-report measures of anxiety and social anxiety. Effects of interpretation bias modification training were found on interpretation bias and social anxiety, but not on attention bias and anxiety measured by the DASS. I have several comments to improve the manuscript.

1) The general aim of this manuscript was to test the efficacy of interpretation bias modification using Chinese word stimuli. However, the importance of this contribution is not well discussed in the discussion.

- Answer:

This contribution was added in the last sentence of the first paragraph in discussion section.

2) What do the authors mean by “benign interpretations”? I think positive would be more appropriate, unless I’m mistaken.

- Answer:

During intervention, a benign interpretation or a negative interpretation word was showed and followed by an ambiguous sentence. “Benign” was used because some of the interpretation word was positive and some of them was neutral.

3) The authors use the word “prove” quite liberally when discussing research findings. This should be changed.

- Answer:

Thank you for the advice. All the “prove” has been replaced.

4) In addition to these wording issues, there are several other locations where the readability in English could be improved.

- Answer:

Language of the manuscript was polished carefully.

5) The authors state in the methods that “Then, participants coded each of their sentence completion as positive, negative or neutral…” could this effect the results? In the context of the training, it may prime certain responses or increase the likelihood of demand characteristic.

- Answer:

Thank you for the advice. We added the influence of demand characteristic as a limitation.

6) On page 8, the authors state “Independent t-tests revealed that there were no differences between ICC and IMP in production of positive and negative interpretations before training (ps>0.05)…” Can the authors provide the full statistics for positive and negative interpretations?

7) Similarly, on page nine when reporting the attention bias effects, the authors state “However, the interaction effect and the main effect of group were not significant (ps>0.05).” Can the full statistics for these effects (and others that may be missing) be reported?

- Answer:

We have revised the results section and related statistics were reported.

8) The authors should briefly discuss why they think there was main effect of time on AB?

- Answer:

We think that as a variable calculated based on reaction time, the practice effect might lead to the change in AB in participants. This was discussed in the third paragraph of discussion section.

9) What was the power for the mediation tests?

- Answer: For the current sample size, Power of the regression tests were 0.67, for medium effect size and two-sided 5% significance level. However, as the mediation tests did not show any significant mediation effect, this result was not added in the manuscript.

Again, thank you very much for the helpful advice. We revised the manuscript carefully following your advice and we hope that the revised manuscript could meet reviewers’ as well as the Journal’s requirement.

The authors
