# Supplementary material for: Efficacy of the Chinese version interpretation bias modification training in an unselected sample: A randomized trial
Source: PLoS One. 2021 Jul 28;16(7):e0255224. doi: 10.1371/journal.pone.0255224 (PMC8318276; doi:10.1371/journal.pone.0255224)
Supplement: Attachment — Submitted filename: response to reviewers-06.docx [file pone.0255224.s006.docx]

**Response to reviewers**

Dear editors and reviewers,

Thank you for your helpful suggestions! We revised the original paper carefully according to the following suggestions. The revised parts were marked in red in the related manuscript. And here are the reviewers’ comments and our reply.

**Reviewer #1:** 
The authors addressed all my previous queries. Maybe, it will be a bit more prudent to provide a reference, or some references, to the use of reciprocals for normalization, as that is not so commonly used!

- Answer:

Thank you for the advice. The methodology of Box and Cox transforms [1] was used widely to achieve normal distribution [2,3]. The family of transformations for Y > 0 was of the form:

λ was the parameter of the transformation. This family of transformations included most of those in use by statisticians such as the square root, log transform, etc. If λ=-1, Y(λ) would equal to the reciprocals. In our study, applying the Box–Cox transformation with λ=-1 allowed obtaining a Shapiro–Wilk coefficient. Z=0.11, p=0.16 for reciprocal of DASS_A before training, and Z=0.11, p=0.09 for reciprocal of DASS_A after training.

Reference

1. Box, G.E.P. and Cox, D.R. (1964). An Analysis of Transformations. Journal of the Royal Statistical Society: Series B (Methodological), 26: 211-243. doi:10.1111/j.2517-6161.1964.tb00553.x

2. Casal C.A., Anguera M.T., Maneiro R., Losada J.L. (2019). Possession in Football: More Than a Quantitative Aspect – A Mixed Method Study. Front Psychol,18;10:501. doi:10.3389/fpsyg.2019.00501.

3. Gecili E.，Ziady A.，Szczesniak R.D. (2021). Forecasting COVID-19 confirmed cases, deaths and recoveries: Revisiting established time series modeling through novel applications for the USA and Italy. PLoS One. 2021,16(1):e0244173. doi: 10.1371/journal.pone.0244173.

**Reviewer #2:**

Thank you to the authors for revising their manuscript following my comments. Although the revisions were largely helpful, there were some points where something was still unclear or potentially problematic:

2.5 Sample size calculation:

This still needs a bit more information, e.g. what kind of interaction (incl. how many groups and time-points), and what kind of effect size (partial eta squared?) is the 0.4? And why was a large effect assumed?

- Answer:

Thank you for the advice. Sample calculation was modified as:

G*Power software version 3.1.9.2 was used to estimate required sample size for this study. This study used repeated measurement analysis of variance (ANOVA) to analyze time, group and interaction effects of the interventions. Therefore F-test (ANOVA repeated measurement, within-between interactions) was chosen. Sample size was based on detecting changes in social anxiety levels. In line with the previous study[1], effect size (η_p_^2^) was set at 0.23 and alpha value was set at 0.05. Approximately 40 participants (i.e., 20 participants in each arm) would provide 80% power and 52 participants (i.e., 26 participants in each arm) would provide 90% power to detect a statistical significance.

Here, the power of 80%[2] or 90%[3] has both been widely used and we provide the results of sample calculation under both conditions.

References:

1 Amir, N., & Taylor, C. (2012). Interpretation Training in Individuals with Generalized Social Anxiety Disorder: A Randomized Controlled Trial. J consult clin psycho, 80, 497-511. doi: 10.1037/a0026928

2 Antognelli SL, Sharrock MJ, Newby JM. (2020). A randomised controlled trial of computerised interpretation bias modification for health anxiety. J Behav Ther Exp Psychiatry, 66:101518. doi: 10.1016/j.jbtep.2019.101518.

3 Moore B, Dudley D, Woodcock S. (2019). The effects of martial arts participation on mental and psychosocial health outcomes: a randomised controlled trial of a secondary school-based mental health promotion program. BMC Psychol, 7(1):60. doi: 10.1186/s40359-019-0329-5.

I think the authors have misunderstood this item on the CONSORT checklist- this is about the whole trial (e.g. stopping if many adverse events noticed) not individual participants.

- Answer:

Thank you for the advice. We relearned the CONSORT checklist and found that the “interim analyses and stopping guidelines” was not applicable for the present study. We are very grateful to the reviewer for reminding us of use of stopping guidelines.

2.7 Randomization and blinding

There is still not enough information about the randomization - how were these numbers generated by the computer? Or were the numbers just sequential, alternating odd and even, in which case the allocation is not really random (which would be quite a major limitation that would need addressing in the discussion).

"Allocation concealment was kept until the end of the study" - I think the researchers have misunderstood the term allocation concealment and should read the CONSORT elaboration document carefully.

- Answer:

The random number (ranged from 1 to 100) was generated by the Microsoft Excel using the random number function. This detailed information was added in the manuscript.

According to CONSORT, allocation concealment seeks to prevent selection bias. But in the current study, post-training tests were self-rating scales which would be influenced by demand characteristics of the participants. As a result, participants and the experimenter did not know group assignment during intervention assignment and pre-/post-training tests. In line with the CONSORT checklist as well as the reality, we revised the sentence to “Allocation concealment was kept until interventions and post-training tests were finished.”

2.8 Data analysis

It is good to have some extra information about the multiple imputation, but I am not sure I fully understand - the authors say all dependent variables were included - does this mean only post-training variables, or also baseline variables? It would be good to be more explicit about this in the paper.

- Answer:

As shown in Figure 1, one participant forgot to take post-training tests of SCT, AB and IAS and his missing data were generated by multiple imputation. The rest of the data were complete. See 2.8 Data analysis for the modified parts.

Some minor points:

The sentence in the abstract "But few Chinese training procedure was available and the effect of interpretation training on attentional bias remained unclear." would read better as "However, little research had been carried out in Chinese samples, and the effect of interpretation training on other processes such as attentional bias also remained unclear.

And in the second to last sentence it would be better to say 'efficacy' rather than 'effectiveness' of the training.

In the section 2.3 Intervention

"Participants would saw a total..." should be "Participants saw a total"

In 2.8 Data analysis

" skewers distribution" should be "skewed distribution"

- Answer:

Thank you for the advice. The sentences and words were revised and marked in the manuscription.

Again, thank you very much for the helpful advice. We revised the manuscript carefully following your advice and we hope that the revised manuscript could meet reviewers’ as well as the Journal’s requirement.

The authors
